# Treatment of Anorexia Nervosa—New Evidence-Based Guidelines

**DOI:** 10.3390/jcm8020153

**Published:** 2019-01-29

**Authors:** Gaby Resmark, Stephan Herpertz, Beate Herpertz-Dahlmann, Almut Zeeck

**Affiliations:** 1 Department of Psychosomatic Medicine and Psychotherapy, University Hospital Tuebingen, Osianderstr. 5, 72076 Tuebingen, Baden-Wuerttemberg, Germany; 2 Department of Psychosomatic Medicine and Psychotherapy, LWL University Hospital, Ruhr-University Bochum, Alexandrinenstr. 1-3, 55791 Bochum, Nordrhein-Westfalen, Germany; stephan.herpertz@rub.de; 3 Department of Child and Adolescent Psychiatry, Psychosomatics and Psychotherapy, University Hospital of the RWTH Aachen, Neuenhofer Weg 21, 52074 Aachen, Nordrhein-Westfalen, Germany; bherpertz@ukaachen.de; 4 Department of Psychosomatic Medicine and Psychotherapy, University Hospital Freiburg, Hauptstr. 8, 79104 Freiburg, Baden-Wuerttemberg, Germany; almut.zeeck@uniklinik-freiburg.de

**Keywords:** anorexia nervosa, guidelines, evidenced-based, treatment

## Abstract

Anorexia nervosa is the most severe eating disorder; it has a protracted course of illness and the highest mortality rate among all psychiatric illnesses. It is characterised by a restriction of energy intake followed by substantial weight loss, which can culminate in cachexia and related medical consequences. Anorexia nervosa is associated with high personal and economic costs for sufferers, their relatives and society. Evidence-based practice guidelines aim to support all groups involved in the care of patients with anorexia nervosa by providing them with scientifically sound recommendations regarding diagnosis and treatment. The German S3-guideline for eating disorders has been recently revised. In this paper, the new guideline is presented and changes, in comparison with the original guideline published in 2011, are discussed. Further, the German guideline is compared to current international evidence-based guidelines for eating disorders. Many of the treatment recommendations made in the revised German guideline are consistent with existing international treatment guidelines. Although the available evidence has significantly improved in quality and amount since the original German guideline publication in 2011, further research investigating eating disorders in general, and specifically anorexia nervosa, is still needed.

## 1. Introduction

### 1.1. Anorexia Nervosa

Anorexia nervosa (AN) is a serious illness leading to high morbidity and mortality [1,2,3,4]. It is characterised by a restriction of energy intake, weight loss, fear of weight gain and distorted body image. According to the diagnostic criteria of the International Classification of Diseases, 11th Revision (ICD-11) [5] and the Diagnostic and Statistical Manual of Mental Disorders, 5th Edition (DSM-5) [6], the resulting malnutrition and low body weight may result in massive impairment to health. Often it takes years for patients with AN to achieve a first remission or to recover permanently. A quarter of adult patients go on to develop an enduring form of the disorder, and one-third of patients continue to suffer from residual symptoms in the long-term. The long-term outcome of adolescent-onset AN is more favourable [7]. Because of its severe and protracted course, AN represents a high emotional and economic burden for sufferers, carers and the society in general [8,9]. Age of onset peaks in middle to late adolescence, which affects educational and professional development. The consequences of starvation can have a negative impact on bone density, growth, and brain maturation, especially in children and adolescents. Many patients are affected by comorbid psychological diseases, such as depression, anxiety or obsessive–compulsive disorder. Additionally, the ego-syntonic nature of AN leads to a strong ambivalence regarding weight gain and recovery, which complicates and often slows down the recovery process. In light of these factors, treatment of AN remains challenging. To improve patients’ chances of recovery, all individuals dealing with this illness should be well informed about the nature and challenges of treating AN. 

### 1.2. Evidence-Based Treatment Guidelines for Eating Disorders

Evidence-based guidelines have been developed in several countries around the world to guide the treatment of different eating disorders, such as AN. These guidelines have the following aims [10]:

- To support all professionals involved in the diagnosis and treatment of eating disorders, as well as sufferers and their relatives, in deciding on adequate measures of care (prevention, diagnosis, therapy and aftercare);
- To improve health care outcomes;
- To minimise risks;
- To increase treatment safety and efficiency;
- To avoid non-indicated diagnostic and treatment methods.

Further, guidelines can reveal gaps in the health care system [11] and inspire new paths of research. 

Treatment guidelines provide recommendations based on current scientific evidence. In cases where a lack of scientific evidence is available, recommendations are often provided based on expert opinion, influenced by years of clinical experience.

## 2. The German S3Guideline for the Diagnosis and Treatment of Eating Disorders

### 2.1. Historical Development of the S3-Guideline

In 2000, the German Society for Psychiatry, Psychotherapy and Psychosomatics (DGPPN) published a guideline for the diagnosis and treatment of eating disorders in Germany for the first time [12]. In the same year, a guideline of the German Society of Child and Adolescent Psychiatry, Psychosomatics and Psychotherapy (DGKJP) was also published [13]. Both guidelines were developed by expert groups using informal consensus (a representative group of experts from the relevant medical society prepares a recommendation which is adopted by the board of the society, development stage one) with the aim of developing recommendations for the diagnosis and treatment of eating disorders. In the autumn of 2003, a conference of members of the German Society for Psychosomatic Medicine and Medical Psychotherapy (DGPM) and the German College of Psychosomatic Medicine (DKPM) decided to develop an evidence-based guideline for eating disorders in Germany according to development stage three (S3, based on all elements of systematic development—logic, decision and outcome analysis, evaluation of the clinical relevance of scientific studies and periodic review).

One year later, in the spring of 2004, a group composed of psychiatrists, child and adolescent psychiatrists, medical specialists in psychosomatic medicine and psychologists with expertise in eating disorders, was formed. The group included representatives of the five professional societies (DGKJP, the German Psychological Society (DGPs), DGPM, DGPPN and DKPM) that are responsible for the care of patients with eating disorders within the German health care system. In 2010, the evidence-based guideline for the diagnosis and treatment of eating disorders was published online by the Association of the Scientific Medical Societies in Germany (AWMF) [14]. The AWMF advises on matters and tasks of fundamental and interdisciplinary interest in medicine and provides, among other things, a wide range of clinical practice guidelines on its website. The AWMF is the national member for Germany in the Council for International Organisations of Medical Sciences (CIOMS) at the World Health Organisation, Geneva. In 2011, the guideline was made available in book format [15]. Based on the scientific guideline, a patient guideline was published in 2015 [16]; this guideline, supported by the German Society for Eating Disorders (DGESS), was designed to communicate the content of the scientific guideline to patients and relatives. The patient guideline, available both online [16] and in book format [17], addresses care structures and supports communication with professional health care providers, such as the family doctor, medical or psychological psychotherapists for adults or child and adolescent psychiatrists and psychotherapists.

Over the last two years, the scientific guideline has been revised, and a second edition will be available in German at the beginning of 2019, both online [14] and in book format. An English version of the guideline is currently in preparation and will be released at a later date. The scientific guideline addresses all age groups and is available in both a short and an extended version. The thematic structure of the recent guideline largely corresponds to the first edition and includes chapters covering epidemiology, diagnostics, the therapeutic relationship, AN, Bulimia nervosa (BN), Binge eating disorder (BED), physical sequelae and methodology. The chapter ‘Diagnostics’ is subdivided into sections on the diagnostics of psychological and somatic symptoms. In line with DSM-5 [6], two new categories of eating disorders have been added to the revised guideline: the ‘Other Specified Feeding or Eating Disorders’ (OSFED), which also include the ‘Night Eating Syndrome’, and the ‘Avoidant Restrictive Food Intake Disorder’ (ARFID), which replaces the old category of ‘Eating Disorders Not Otherwise Specified’ (EDNOS). With regard to the therapeutic studies on AN [18], BN [19] and BED [20], meta-analyses were performed based on a systematic literature search and assignment of pre-determined quality indicators (evidence level I). 

### 2.2. Recommendation for AN—Differences between the First Version and the Revision

Changes in treatment recommendations were based on a systematic literature search (2008–2017), in which 26 new randomised controlled trials (RCTs) on psychotherapeutic treatments, 13 new RCTs on pharmacotherapy and 2 new RCTs on nutritional management were identified [14,18]. The evidence base has considerably improved since the first version, although studies still show a large heterogeneity in terms of samples (adolescents, adults, severe and enduring AN), setting (outpatient, day hospital, inpatient), treatment phase (acute, maintenance) and outcome measures used. It should be emphasised that an improvement in study quality can be seen. In recent years, for example, studies have been published with sample sizes that allow sufficient statistical power [21,22,23,24,25,26].

Treatment recommendations were based on a network-meta-analysis (see Section 2.3), newly published RCTs, systematic reviews, or lower levels of evidence (if RCTs or systematic reviews were not available). The guideline group discussed each recommendation in light of the available evidence, clinical relevance and suitability. The most relevant changes in the revised version concerning evidence levels and recommendations are summarised in Table 1. Evidence levels were assigned using the Oxford Centre of Evidence Based Medicine criteria [27]: An evidence level of I is given if there is evidence for a specific treatment based on a systematic review (or meta-analysis) of randomised controlled trials (Ia), or one randomised controlled trial with narrow confidence interval (Ib). An evidence level of II is based on cohort-studies (IIa: systematic review, or IIb: individual cohort study). Evidence level III refers to case-control studies and evidence level IV to case-control series. 

Treatment recommendations in the German treatment guideline were graded according to levels ‘A’, ‘B’, ‘0’ and ‘KKP’ [28]. ‘A’ is the strongest recommendation, which is usually based on evidence level I (something ‘is to be done’). ‘B’ recommendations are less strong (something ‘should be done’; evidence level II) and ‘0’ recommendations are even less explicit (something ‘may be done’). ‘KKP’ (‘clinical consensus point’) stands for recommendations, which are not based on empirical research and were derived from the experience of experts (good clinical practice). Grading of recommendations was based largely on the evidence level, but also took the following criteria into account: clinical relevance of effect sizes and end points, the balance of benefits and risks, ethical considerations, patient preferences and applicability. Grading of recommendations was discussed in several consensus meetings.

Several key treatment recommendations did not change. They will be referred to in the comparison of evidence-based guidelines from other countries (see Section 3.2). 

Up and down-grading of recommendations: Only one recommendation was downgraded. It is the recommendation for the use of low-dose neuroleptics in some cases of AN. The decision was based on the consideration that this recommendation should be followed only with caution and not as an overall clinical standard. In contrast, the recommendation not to use neuroleptics for the treatment of AN was upgraded due to an increase in evidence (systematic reviews). The same is true for the recommendation to continuously address motivation to change throughout treatment. Several studies show that motivation to change is a relevant predictor of treatment outcome. Recent high-quality trials made it possible to make specific recommendations regarding the use of specialised psychotherapeutic treatments. However, due to ethical reasons, no study compared an active treatment with untreated control groups. Therefore, it was decided that the recommendation should be classified as ‘B’ and not ‘A’. A further recommendation was upgraded based on clinical relevance; Inpatient treatment should take place in facilities which are able to offer a specialised multimodal treatment programme. In Germany, some adult and child and adolescent psychiatric and psychosomatic hospitals are not specialised and have no experience with the treatment of patients with AN. Treatment in such facilities is, therefore, not recommended, due to high associated risks, not to mention high costs. The new guideline also includes the explicit recommendation to consider co-morbidity in patients with AN. Co-morbid conditions like borderline-personality disorder or post-traumatic stress disorder, for example, might require changes in treatment planning and prioritisation of therapy goals. Although empirical evidence is scarce, a recommendation for a stabilisation phase as a final phase in inpatient treatment was included, as relapse after discharge is common [29,30,31], and the transition from one service level to another service level (especially to a level with less supervision and support) is a major challenge for patients with AN. Finally, there was new empirical evidence suggesting that a short inpatient stay for weight stabilisation followed by day hospital treatment is as effective as long-term inpatient treatment for children and adolescents with AN, providing there is continuity in the therapists that are responsible and if there is sufficient support by family members [23].

### 2.3. Network-Meta-Analysis

Based on the systematic literature search (see Section 2.2), a network-meta-analysis was conducted to answer the following question: What is the comparable effectiveness of different psychotherapeutic treatments for AN? Additionally, two further questions were addressed using standardised mean change statistics: What is the amount of weight gain that can be expected in different treatment settings? And: What is the amount of weight gain that can be expected in adolescents vs. adults?

Predefined inclusion and exclusion criteria were used to select the studies. Each study was rated by two independent researchers and additionally assessed for quality [18]. For more details on data analysis see [18]. 

Network-meta-analysis: 18 randomised controlled studies met inclusion criteria for the data-analysis. Ten studies were on adolescents (625 patients), and 8 studies were on adults (622 patients). No treatment approach was found to be superior. However, there were several limitations to the analysis and interpretation of results. All studies compared active treatments with each other, with no study including an untreated control group. Only a few comparisons were replicated. Furthermore, the majority of studies on adolescents evaluated family-based treatment approaches mostly by the same group of researchers, while interventions in adults were almost exclusively on an individual basis. The manualised treatment approaches that were evaluated in high quality trials comprise the Maudsley Model of Anorexia Nervosa Treatment for Adults (MANTRA) [25], Focal Psychodynamic Therapy (FPT) [26,32,33], Enhanced Cognitive Behaviour Therapy (CBT-E) [26,34,35], Specialist Supportive Clinical Management (SSCM) for adults [25,35,36], and family-based treatment (FBT) for adolescents [21,22,24]. 

Standardised mean change statistics (SCM): Analyses were conducted with 38 studies (1164 patients). Seventeen of these studies were naturalistic studies, and four studies were on adolescents (350 patients). For a course of up to 27 weeks, significantly higher weight gains can be expected in inpatient treatment compared to outpatient treatment (for adults: mean weight gain of 537 g/week in inpatient treatment vs. 105 g/week in outpatient treatment; for adolescents: mean weight gain of 615 g/week in inpatient treatment vs. 192 g/week in outpatient treatment). The estimated effect sizes for weight gain in adolescents were significantly higher compared to adults (in RCTs: SMC = 1.97 vs. 1.02, in naturalistic studies SMC = 1.84 vs. 1.42, respectively).

In sum, there are several existing manualised psychotherapeutic treatments for AN, which can be considered evidence-based and effective. However, there is a need for replication studies. There are differences regarding treatment response and most suitable treatment approach in adult versus adolescent patients.

## 3. Comparison of the German S3-Guideline with International Evidence-Based Clinical Treatment Guidelines

### 3.1. International Evidenced-Based Eating Disorders Guidelines

There are currently several additional evidence-based guidelines available, which provide recommendations regarding the diagnosis and treatment of eating disorders. Most of the guidelines were written by multidisciplinary groups (comprising health care professionals and researchers), and most were designed solely for use by health specialists involved in the treatment of eating disorders. The most recent of these guidelines are the Dutch [37] and the revised British guidelines [38], both published in 2017. The British guideline [38], published by the National Institute for Health and Care Excellence (NICE), addresses all age groups (children, adolescents and adults), and all eating disorder categories (AN, BN, BED and Other Specified Feeding or Eating Disorders (OSFED)). Several lay members of the community were involved in the development of this guideline. The Dutch guideline addresses AN, BN and BED [39]. This guideline, designed to be used by both specialists and population members, is only available in Dutch [39]. Healthcare professionals collaborated with patients and relatives, as well as health insurance representatives, during the developmental stages of the guideline [39]. 

The next most recent guideline, published in 2016, is the Danish guideline [40]. This ‘quick guide’, provides a brief overview, designed solely for the treatment of AN. The guideline is available in English, and it addresses all age groups. The full-length version of this guideline is only available in Danish. The Australian and New Zealand guideline [41] was published in 2014 by the Royal Australian and New Zealand College of Psychiatrists. Community members and stakeholders collaborated with healthcare professionals and academics in the development of the guideline. This guideline contains two sections separately addressing AN in children and adolescents, and in adults. BN and BED, as well as avoidant/restrictive food intake disorder, are also addressed. 

In 2012, the American Psychiatric Association (APA) released a guideline watch [42], reviewing new evidence published since the last APA guideline in 2006, but gives no explicit recommendations [43]. This guideline addresses AN, BN and BED, and also makes reference to EDNOS. The guideline is designed primarily for the treatment of adults, but also briefly addresses the treatment of children and adolescents. The French guideline [44], published in 2010, is written specifically for AN. It addresses all age groups and is available in English. In 2009, the Spanish guideline [45] for eating disorders was published. This guideline, which concerns eating disorder patients over 8 years of age, is written not only for healthcare specialists, but also for the population and educational professionals. It addresses AN, BN, BED and EDNOS. 

In addition to these national guidelines, several more specific evidence-based guidelines also exist. A guideline, developed specifically for the Canadian province of British Columbia, was released in 2010 [46]. This guideline addresses AN, BN and EDNOS (except BED), and advisesthe on treatment of all age groups. In 2011, the World Federation of Societies of Biological Psychiatry (WFSBP) released a guideline specifically addressing the pharmacological treatment of eating disorders [47]. This guideline, written in English, addresses the pharmacological treatment of AN, BN and BED. In 2014, the Management of Really Sick Patients with Anorexia Nervosa (MARSIPAN and Junior MARSIPAN) guideline [48] was published, a guideline which specifically addresses the treatment of children, adolescents and adult patients with ‘severe’ AN. 

In line with an evidence-based approach, most of the guidelines explicitly state that the development of the guideline involved a systematic literature review, a rating of the identified literature, and a complex consensus process, involving collaboration and review by numerous experts [14,38,41,43,44,45,46,47,48]. Only the MARSIPAN [48] and WFSBP guidelines [47] do not explicitly refer to a complex consensus process, and the British Columbia guideline [46] does not mention a rating system. The Danish ‘quick guide’ [40] has a complete absence of information on the methodological process. However, the inclusion of evidence levels in the guide implies that the developmental process was rigorous. A detailed review of the evidence upon which the recommendations are based is only available in the British guideline and the Danish full-length guideline. A more detailed comparison of the methods employed in developing the guidelines goes beyond the scope of this review article. All of the guidelines are available online. The Australian and New Zealand, MARSIPAN, WFSBP and APA guidelines are published in online scientific journals and partly in print versions, and the remainder of the guidelines are available on the relevant publishing society’s website.

### 3.2. Commonalities and Differences

#### 3.2.1. Treatment Setting

For adults: Similar to the German guideline [14], all remaining guidelines (excluding the Danish [40] and WFSBP guidelines [47]) recommend outpatient treatment as a first treatment option, suggesting day patient or inpatient treatment as a more intensive treatment option if outpatient treatment proves ineffective [38,39,41,43,44,46,48]. The German guideline states, however, that in some cases this ‘stepped-care’ approach may not be appropriate.

Inpatient treatment is recommended in cases with a BMI <15 kg/m², rapid or continuing weight loss (>20% over 6 months), high physical risk, severe co-morbid conditions or denial of illness. If these criteria are met, an inpatient setting may be necessary for initial treatment. Likewise, all remaining guidelines (excluding the Danish and WFSBP guidelines) also suggest more intense treatment settings from the outset in cases of severe medical instability. All of these guidelines provide information regarding hospital admission criteria with varying degrees of detail, but agree on the necessity to judge the need for hospitalisation on an individual and multifactorial basis. Further, they state that compulsory treatment is possible in the case of extreme medical complications. The Danish and WFSBP guidelines do not make reference to treatment setting. For an overview of indicators of high medical risk and the handling of medical complications see the review of Zipfel and colleagues [4].

For children and adolescents: Corresponding to the treatment recommendations for adults, outpatient treatment is proposed as the first line treatment for children and young people by the German [14] and most other guidelines [38,41,43,44] if the patient is in a stable medical state. If more intensive care is needed, several guidelines suggest a graduated procedure from inpatient to partial and finally to outpatient treatment programs [40,44,45]. Only the German guideline [14] gives a special recommendation for a referral to day patient treatment. Interestingly, the British and accordingly the Spanish guidelines advise admitting children and young people to a setting with age-appropriate facilities, which are near to their home and have the capacity to provide appropriate educational activities [38,45].

Regarding medical risk and necessity for inpatient treatment, the Australian and New Zealand, British Columbia, British, APA and French guidelines [38,41,44,46,49] provide exact criteria, such as a BMI below the 3^rd^ percentile or an expected body weight (EBW) below 75%, an abnormally low heart rate or blood pressure, electrolyte disturbances, etc. However, the exact values vary between countries. As for adults, these guidelines also indicate psychiatric risk factors, such as suicidality or severe self-injurious behaviour. The German and Spanish guidelines [14,45] are more unspecific to indicate hospitalisation (see above). The German and French guidelines [14,44] also refer to psychosocial risks, such as social isolation and family crisis, to consider inpatient treatment.

#### 3.2.2. Psychotherapy

For adults: All guidelines except for the Danish [40] and WFSBP [47] address the efficacy of specific psychological interventions. No guideline recommends one single superior treatment option. The German [14], British [38] and Dutch guidelines [39] conclude that cognitive-behavioural therapy (CBT or CBT-E respectively), MANTRA, and SSCM are equally effective treatment options, and so, all treatments are recommended as first-line options. Additionally, the German guideline recommends FPT as another first-line treatment option. The remaining guidelines all review evidence for CBT, as well as a variety of other treatments including SSCM [41], psychodynamic therapy [43,44,45,46], interpersonal therapy [43,45,46], behaviour therapy [45] and ‘systematic and strategic therapies’ [44]. These guidelines all conclude that psychological interventions are effective, however, state that there is insufficient evidence to identify which is the most efficacious. The French [44], Dutch [39] and APA guidelines [43] also suggest that psychological interventions may not be as effective in severely malnourished patients. 

The Danish guideline [40] also recommends the use of psychotherapeutic treatments, however, does not make any recommendations regarding specific interventions. This guideline provides a ‘weak recommendation’ that both group and individual psychotherapeutic treatment be considered as first-line treatment options, based on ‘very low evidence’ which suggests the approaches are equally effective. Recommendations for the inclusion of alternative elements, such as meal support and supervised physical activity, during the treatment phase are mentioned. Other guidelines make specific recommendations against alternative treatments; for example, the German [14] and the Australian and New Zealand guidelines [41] state that nutritional counselling alone should not be used as the sole treatment, and the British guideline [38] recommends against the use of alternative physical therapies, such as yoga, warming therapy, transcranial magnetic stimulation and acupuncture. The Spanish guideline [45] also advises against the use of excessively rigid behavioural programs for inpatients. 

Some guidelines make recommendations regarding the required duration of treatment. The Australian and New Zealand guideline [41] states that a longer-term follow-up is necessary as relapse is common, and the Spanish guideline [45] states that duration of treatment should span at least six months for outpatients and twelve months for inpatients. The APA guideline [43] states that due to the enduring nature of the illness, psychotherapeutic treatment is usually required for at least one year, and the British guideline [38] makes specific recommendations regarding the time span of treatments, for example suggesting that CBT treatment for eating disorders should consist of 40 sessions over 40 weeks. The French guideline [44] recommends that treatment should last at least one year after significant clinical improvement, and the German guideline [14] states that after outpatient treatment, patients should regularly meet with their general practitioner (GP), or other care coordinator, for at least one year. The German guideline also recommends that the last phase of inpatient treatment before transfer to an outpatient setting should include a stabilisation period where patients demonstrate that they can maintain the achieved weight gain for a specified amount of time. 

Some treatment guidelines make additional specific recommendations. The German [14], French [44], MARSIPAN [48] and Australian and New Zealand guidelines [41] all emphasise the importance of adopting a multi-disciplinary, collaborative approach to treatment. In a similar vein, the German [14], British Columbia [46] and APA guidelines [43] highlight the importance of effective communication between all involved health workers, and recommend identifying someone to act as the primary care coordinator, such as the patient’s GP. 

The MARSIPAN guideline [48] is specifically written regarding the treatment of patients who have a severe or enduring form of AN. The Australian and New Zealand [41] and British Columbia guidelines [46] also include comprehensive sections which address the treatment of such patients and suggest taking an alternative approach, focused on enhancing quality of life. The French [44], German [14] and APA guidelines [43] also briefly mention the treatment of patients with enduring AN. Other guidelines provide information regarding other additional elements related to AN. For example, both the Spanish [45] and French guidelines provide information regarding the care required for pregnant patients. Additionally, the APA and British Columbia guidelines include recommendations for therapists and specialists regarding communicating with patients (for example addressing the therapeutic relationship, boundaries). The German guideline does not entail any recommendations, but devotes a separate chapter to this topic.

For children and adolescents: All guidelines strongly recommend the involvement of parents or near caregivers in all treatment settings. The Australian and New Zealand, Spanish, APA and German guidelines explicitly mention family-based treatment or therapy (FBT) [14,41,43,45]. However, the Australian and British guidelines also propose alternatives if FBT is not appropriate, such as other forms of family therapy [41], as well as individual treatment, such as adolescent-focused therapy (AFT) or CBT, in older adolescents [38,41]. No guideline gives an explicit advice whether conjoint or separate FBT should be conducted. The French guideline does not refer to FBT, but to family therapy in general [44]. The British guideline also does not specifically use the term FBT, but has its own terminology instead (anorexia nervosa-focused family therapy, FT-AN) [38]. Although many key features of this treatment resemble FBT, FT-AN also includes other approaches, such as multi-family therapy, conjoint or separate family therapy and exclusion or inclusion of a family meal, which is a core feature of FBT. The British guideline also requests therapists and staff to be aware of or address carers’ needs [38].

A summary of guidelines’ essential key recommendations regarding psychotherapy for AN is shown in Table 2.

#### 3.2.3. Nutritional Management

For adults: The WFSBP guideline [47] suggests that nasogastric feeding is effective for malnourished patients, however, does not address risks associated with refeeding, or provide any specific nutritional or weight gain recommendations. All remaining guidelines, (excluding the Danish guideline [40]), recommend nasogastric feeding for severely malnourished patients, when oral feeding is not an option [14,38,39,41,43,44,46,48]. These guidelines address the risk of refeeding syndrome, recommending that treatment is administered by experienced staff. The APA guideline [43] recommends nasogastric feeding over parenteral feeding, and the British guideline [38] explicitly recommends against parenteral nutrition. The German guideline also discusses the use of percutaneous endoscopic gastronomy feeding as a potential alternative, when patients will not tolerate nasogastric feeding [14]. The Danish guideline does not provide any recommendations regarding refeeding, nutritional intake or weight restoration. 

In the original German guideline [15], an initial food intake of approximately 30 to 40 kcal/kg per day was recommended for highly underweight patients (see Table 1), which, upon revision, was considered too strict. The revised German guideline [14], as well as the Danish [40], French [44] and WFSBP guidelines [47], do not give specific recommendations regarding energy intake during refeeding. Both the British [38] and MARSIPAN guidelines [48] recommend commencing refeeding at 5 to 10 kcal/kg/day for severely underweight patients, and gradually increasing to 20 kcal/kg/day within 2 days. The British Columbia guideline [46] also recommends beginning refeeding at 5 to 10 kcal/kg/day if severity factors (e.g., nasogastric feeding) are involved. In the absence of severity factors, intake of 20 to 25 kcal/kg/day is recommended, and intake should not exceed 70 to 80 kcal/kg/day. The Spanish guideline [45] recommends a slightly higher caloric intake of 25 to 30 kcal/kg/day for severely malnourished patients, and they also provide a recommended upper limit of 1000 kcal/day. The APA guideline [43] recommends initiating refeeding at 30 to 40 kcal/kg/day, and also suggests that males may require a significantly higher energy intake to gain weight. The Dutch guideline has an even higher recommended refeeding starting point of 40 to 60 kcal/kg/day for severely underweight patients [39]. The Australian and New Zealand guideline [41] does not provide a recommended nutritional intake based on weight, but instead recommends a specific starting intake of 1433 kcal/day, with increases of 478kcal every 2 to 3 days. 

Several guidelines also provide recommendations regarding appropriate weekly weight gain goals in inpatient and outpatient settings. Five guidelines recommend a minimum weight gain of 0.5 kg/week in an inpatient setting; the German [14], French [44] and Spanish guidelines [45] recommend weight gain ranging between 0.5 and 1 kg/week, the Australian and New Zealand guideline [41] recommends weight gain between 0.5 and 1.4 kg/week, and the Dutch guideline suggests weight gain ranging between 0.5 and 1.5 kg/week [39]. In contrast, the British Columbia guideline [46] suggests a higher minimum weight gain ranging from 0.8 to 1.4 kg/week, and the APA guideline [43] suggests a minimum weight gain ranging from 0.9 to 1.4 kg/week. The remaining guidelines [38,40,47,48] do not provide specific weight gain recommendations. Only four of the guidelines provide recommendations regarding weight gain per week in an outpatient setting. The French guideline recommends a weight gain of 0.25 kg/week, while the German, APA guidelines and Dutch recommend a weekly gain of between 0.2 to 0.5 kg [39].

For children and adolescents: The British guideline for the management of severely ill young people with AN (Junior MARSIPAN) [48,50] advocates to commence refeeding at about 40 kcal/kg/day and increase the meal plan by 200 kcal/day, while the others do not explicitly give calorie specifications for children and adolescents. Almost all guidelines recommend nasogastric tube feeding, if a meal plan and supplement drink tops are not managed [14,41,43,45,50].

The French, Danish and German guidelines emphasise the necessity of achieving a target weight at which menstruation can reoccur [14,40,44]. While the French guideline does not give any threshold criteria, the German guideline defines the 25^th^ age-adapted BMI-percentile (with the 10th percentile as a minimum) in contrast to the Danish guideline with the 50^th^ weight-for height percentile as target weight.

Supplementary nutritional counselling is advised by the British, Spanish and German guidelines for children and adolescents and their carers to help young people meet their dietary needs for pubertal development and growth [14,38,45]. According to these guidelines, growth and pubertal development should be regularly monitored in this age group. 

#### 3.2.4. Psychopharmacology

For adults: Use of pharmacotherapy is addressed in all treatment guidelines excluding the Danish guideline [40]. All of these guidelines emphasise the lack of evidence surrounding medication use for AN, and most guidelines emphasise that caution must be taken when administering medication, due to the physical complications associated with AN (e.g., cardiac problems). The Spanish [45], APA [43] and British guidelines [38] explicitly state that medication should not be used as the sole treatment. The British guideline also states that there is no proven benefit of combined treatment over psychotherapy alone in treating patients without comorbidities. All guidelines excluding the MARSIPAN [48], Danish and British guidelines give cautious recommendations for the use of antipsychotic medications. The French guideline [44] provides a cautionary recommendation, without addressing specific medications or effects. The remaining guidelines all make specific reference to the antipsychotic olanzapine; the German [14], WFSBP [47], Dutch [39], Australian and New Zealand [41], and APA guidelines recommended it to assist with anxious and obsessional thoughts, the WFSBP and Spanish guidelines suggest that it may be useful for improving general psychological symptoms, and the British Columbian [46], Spanish and APA guideline cautiously recommended it for improvements in weight gain. In contrast, the German guideline recommends against the use of antipsychotics for weight gain. The German guideline states there is no conclusive evidence to recommend the use of antidepressants for the core symptoms of AN, and the Dutch guideline also explicitly recommends against the use of selective serotonin reuptake inhibitors (SSRIs) [39]. In contrast, antidepressants are cautiously recommended by the French, WFSBP and APA guidelines, to assist with co-occurring symptoms of depression, obsessive–compulsive or anxiety disorder. Specifically, the APA guideline discusses the advantages of using selective serotonin reuptake inhibitors in combination with psychotherapy to address persistent depressive or anxiety symptoms, but recommends against the use of monoamine oxidase inhibitors and bupropion, due to adverse reactions and health risks. The APA guideline cautiously recommends the use of pro-motility agents for use against bloating, and use of antianxiety agents before eating for some patients. Similarly, the MARSIPAN guideline [48] discusses the use of benzodiazepines for particularly anxious patients. The WFSBP and APA guidelines discuss potential weight gain benefits of taking zinc supplements, while the German guideline suggests restricting zinc supplementation to cases with proven zinc deficiency.

For children and adolescents: With the exception of hormone replacement therapy the German and most other international guidelines do not give any specific recommendations for this age group. The Junior MARSIPAN guideline concludes that it ‘may be necessary to prescribe regular sedative antipsychotic medication, such as olanzapine’, if the patients are extremely agitated and resist refeeding [48]. It also gives clear recommendations for ECG monitoring if antipsychotics are applied. Hormone replacement therapy: In several guidelines including the German guideline the prescription of an oral contraceptive is not recommended [38,41,43]. The British guideline suggests considering a bone mineral density scan after one year of underweight in children and adolescents. Moreover—in correspondence to the German guideline—the British guideline suggests to consider transdermal estrogen replacement in combination with cyclic progesterone application in girls with a bone age over 15 years and long-term underweight as well as incremental physiological doses of estrogen in those below 15 years [14,38]. Similar indications are mentioned in the APA and the Australian and New Zealand guidelines [41,43].

## 4. Discussion

This review provides an overview of the newly revised and published German S3-guideline for eating disorders [14]. In particular, it highlights the changes in recommendations regarding the treatment of AN since the publication of the original guideline in 2011 [15]. In summary, family-based therapy approaches are recommended for adolescents, whereas individual approaches are suggested for adults. There is no evidence indicating the superiority of one specialised approach over another. In more intensive settings, as well as in adolescents, higher weight gains can be expected. To date, there is no convincing evidence for the positive effect of pharmacotherapy regarding the core symptoms of AN.

The revised German guideline is currently the most recent eating disorder treatment guideline internationally. Recommendations are, therefore, based on the most up to date research findings and evidence available. The development of this guideline involved a rigorous process, including a comprehensive literature review and analysis, and consultation and contribution by many experts in the eating disorder field. The findings of the literature review and network analysis are also available in English [18]. 

The German guideline also includes an easily comprehensible guide for sufferers with eating disorders and their relatives [17], which has been developed with the help of patient representatives. The German guideline, hereby, stresses the necessity of providing information and support to significant others, who often bear a high emotional burden, but also play an important role in helping patients to overcome the eating disorder. The guideline has been published in two different formats—as a scientific book (only the original version so far) and on the website of the Association of the Scientific Medical Societies in Germany (AWMF, awmf.org [14]), where it is freely available. 

Similar to the Dutch guideline, the original version of the German guideline has been published in German only, which limits its distribution and implementation to Germany, Austria and Switzerland. An English translation of the revised version, which is currently in preparation, is, therefore, an invaluable step towards increasing the utility of this guideline. 

The review also explores the similarities and differences between the German guideline and other existing international guidelines. There is significant homogeneity among the international guidelines in the recommendations derived from the existing evidence. All agree that there is no superior treatment for AN, if specialised approaches are compared. There are, however, some inconsistencies regarding aspects, such as medication and nutritional management. Most guidelines implemented a thorough methodology. We think there is a need for European research initiatives which aim to enhance the evidence base and clinical guidance regarding AN across the different participating countries. Recommendations must, however, take into account the specificities of the national health care systems.

Overall, evidence for treatment of AN has increased, yet even in the latest German guideline, many of the recommendations are still based on expert opinion. Guidelines do not only mirror the current state of research but also point out gaps that need to be bridged. There is still a need for more research in the field of eating disorders, particularly in AN. In view of the so-called ‘research-practice gap’, it needs to be mentioned that guidelines are not designed to propagate conformist standard therapy, or to restrict clinicians’ individual willingness to learn and innovate. They should not be seen as directives, but as advice.

## 5. Conclusions

The German S3-guideline is, at present, the most recently revised evidence-based treatment guideline for AN. Based on newly available evidence, several amendments have been made regarding treatment recommendations, since the original guideline publication in 2011. Overall, the recommendations provided in the German guideline are fairly consistent with those provided in other international evidence-based eating disorder guidelines. Adult and adolescent patients should be distinguished in terms of treatment response and the most suitable treatment approach. Although the existing guidelines provide a sound base of information, which can be used by healthcare professionals to guide diagnosis and treatment decisions, further research regarding the treatment of AN is still urgently needed.

## Figures and Tables

**Table 1:** German guideline—changes in treatment recommendations for AN.

Original guideline recommendations 2010 [15]	Guideline-revision recommendations 2019 [14]
General recommendations	
No recommendation concerning co-morbid conditions	(Evidence level IV; Clinical consensus point: good clinical practice): Co-morbid conditions should be systematically assessed and taken into consideration when treating patients with AN.
Treatment setting	
(Evidence level IV; 0): Inpatient treatment should take place in facilities able to offer a specialised multimodal treatment program.	(Evidence level IV; A): Same recommendation as original guideline, recommendation grading updated to A.
No recommendation concerning a stabilisation phase	(Evidence level IV; B): In order toreduce the probability of relapse, the final stage of inpatient therapy should aim to ensure that patients at least maintain their weight for a certain period and are prepared for the transition to an outpatient setting.
No specific recommendation concerning day hospital treatment for children and adolescents	(Evidence level Ib; A): A transfer to day hospital treatment after short-term inpatient treatment with sufficient physical stabilisation should be considered for children and adolescents, provided eating disorder-specific day hospital treatment can be carried out by the same treatment team, and close involvement of the relatives is ensured (evidence level Ib; A).
Psychotherapy	
(Evidence level II; B): Patients with AN are highly ambivalent towards change. Addressing ambivalence and motivation to change is a central task and should be maintained throughout the whole treatment process.	(Evidence level Ia; A): Same recommendation as original guideline, recommendation grading updated to A.
(Evidence level II; B): The outpatient treatment of first choice for AN should be evidence-based psychotherapy. (Clinical consensus point: good clinical practice): Patients with AN should be offered specialised therapy by a practitioner experienced with eating disorders. The choice of method should take into account the patient’s preference and age.	(Evidence level Ib; B): Outpatient treatment of first choice for patients with AN should be evidence-based psychotherapy (FBT for children and adolescents; FPT, CBT-E, MANTRA or SSCM for adults), administered by practitioners experienced with eating disorders.
Nutritional management	
(Evidence level: not rated; statement): For orientation during the first days of treatment, the initial food intake (for enteral nutrition) of highly underweight patients can be quantified at approx. 30–40 kcal/kg.	(Evidence level IIa; statement): In patients with mild to moderate AN, an initial low caloric energy supply with gradual increase is not required for safe weight gain (avoidance of refeeding syndrome)—provided that medical monitoring is ensured.
No recommendation, but formulation of statements. For example, The basal metabolic rate is initially low and increases significantly with the onset of weight gain. The formulas for calculating basal metabolic rate obtained from normal and overweight people are not suitable for use with AN.	(Evidence level IV; Clinical consensus point: good clinical practice ): The energy supply for the expected weight gain is highly variable and should be individually tailored to the patients as well as to the treatment phase and be continuously monitored.
Pharmacotherapy	
(Evidence level Ib; B): Neuroleptics are not suitable for achieving weight gain in AN. (Evidence level Ia; A): Antidepressants are not recommended for achieving weight gain in AN. This applies to both initial therapy and relapse prevention.	(Evidence level Ia; A): Same recommendations as original guideline, recommendation grading regarding neuroleptics updated to A.
(Evidence level IIa; B): If thinking is considerably restricted to weight phobia and eating and if hyperactivity is not controllable, an attempt to use low-dose neuroleptics (especially olanzapine) may be justified in individual cases. The indication for treatment should be limited to the duration of the symptoms mentioned above (no long-term treatment) and should only be applied within the framework of an overall treatment plan.	Same recommendation as original guideline, with altered recommendation levels: (Evidence level IIa; downgrading to 0): If thinking is considerably restricted to weight phobia and eating and if hyperactivity is not controllable, an attempt to use low-dose neuroleptics (especially olanzapine) may be justified in individual cases. (Clinical consensus point: good clinical practice): The indication for treatment should be limited to the duration of the symptoms mentioned above (no long-term treatment) and should only be applied within the framework of an overall treatment plan. The patient must be informed about the circumstances of the off-label use.

FBT, Family-Based Treatment; FPT, Focal Psychodynamic Therapy; CBT-E, Enhanced Cognitive Behaviour Therapy; MANTRA, Maudsley Model of Anorexia Nervosa Treatment for Adults; SSCM, Specialist Supportive Clinical Management.

**Table 2:** International guidelines’ key recommendations regarding psychotherapy for AN.

Recommendation	AUS [41]	BC [46]	DEN [40]	FR [44]	GER [14]	NETH [39]	SP [45]	UK [38]	US [43]
For adults:									
Psychotherapy in general	+	+	+	+	+	+	+	+	+
Not as efficient in severely malnourished patients	N.R.	N.R.	N.R.	✓	N.R.	✓	N.R.	N.R.	✓
Specific psychological interventions	✓	✓	N.R.	✓	✓	✓	✓	✓	✓
CBT/CBT-E	+	+	N.R.	+	+	+	+	+	+
Psychodynamic Therapy	N.R.	+	N.R.	+	+	N.R.	+	N.R.	+
FPT	N.R.	N.R.	N.R.	N.R.	+	N.R.	N.R.	N.R.	N.R.
MANTRA	N.R.	N.R.	N.R.	N.R.	+	+	N.R.	+	N.R.
SSCM	+	N.R.	N.R.	N.R.	+	+	N.R.	+	N.R
IPT	N.R.	+	N.R.	N.R.	N.R.	N.R.	+	N.R.	+
For children and adolescents:									
Involvement of parents/near caregivers	+	+	+	+	+	+	+ ^1^	+	+
Family therapy	FBT or other forms of family therapy	FBT	FBT or other forms of family therapy	Family therapy	FBT or other forms of family therapy	FBT?	Family therapy (systemic or not)	FT-AN	FBT or other forms of family therapy

✓ recommendation given; + explicit recommendation in favour; N.R., no recommendation reported; AUS, Australia and New Zealand; BC, British Columbia; DEN, Denmark; FR, France; GER, Germany; NETH, The Netherlands; SP, Spain; UK, United Kingdom; US, United States; CBT(-E), (Enhanced) Cognitive Behaviour Therapy; FPT, Focal Psychodynamic Therapy; MANTRA, Maudsley Model of Anorexia Nervosa Treatment for Adults; SSCM, Specialist Supportive Clinical Management; IPT, Interpersonal Therapy; FBT, Family-Based Treatment/Therapy; FT-AN, AN-focused Family Therapy; ^1^ and siblings; ?, ambiguous evidence.
